# The lead ores circulation in Central China during the early Western Han Dynasty: A case study with bronze vessels from the Gejiagou site

**DOI:** 10.1371/journal.pone.0205866

**Published:** 2018-11-07

**Authors:** Dian Chen, Wugan Luo, Qingshuo Zeng, Benxin Cui

**Affiliations:** 1 Department of Archaeometry, University of Chinese Academy of Sciences, Beijing, China; 2 Key Laboratory of Vertebrate Evolution and Human Origin of Chinese Academy of Sciences. Institute of Vertebrate Paleontology and Paleoanthropology, Beijing, China; 3 Institute of Cultural Relic Research of Nanyang City, Nanyang, Henan, China; Xiamen University, UNITED STATES

## Abstract

This study first publishes lead isotope data of bronze vessels from Central China in Western Han Dynasty and attempts to find out the lead ores circulation of this time by taking bronzes from the Gejiagou site (Nanyang City, Henan Province) as an example. The elemental concentrations suggest the lead should be introduced on purpose and indicate the provenance information of lead ores. All the lead isotope ratios conform to the characteristics of common lead and most of them are similar to Nanyang local lead ores. The lead of another two bronzes, NY9 and NY13, should be imported from southern China. Combined with the historical background of early Western Han Dynasty, the wider range of the lead ore circulation may be an indicator for, as the loose policy, economic prosperity and transportation improvement.

## Introduction

In the three dynasties of Xia, Shang and Zhou, bronze vessels had an extraordinary role. Because of ritual laws and their values, bronze vessels were used only by the upper aristocracy and given some hierarchical significances [1]. After the Eastern Zhou Dynasty, the rapid development of iron technology had also fostered the progress of bronze industry. Rules of etiquette gradually collapsed. The use of bronze vessels extended from the upper aristocracy to the lower classes of aristocracy. The practical performance of bronze vessels strengthened while their ceremonial natures gradually subsided [1, 2]. But using bronze vessels as the funerary objects has not disappeared and been carried over into the Western Han Dynasty. Therefore, bronze vessels made up the majority of metal vessels all the way through the Later Han period [3].

However, the utilization of bronze vessels at the end of the Bronze Age in China did not get enough attention. Previous studies have shown that the lead isotope ratios of bronze vessels in early Spring and Autumn period (770-476BC) were highly concentrated and metal resources were allocated by the central authority. Entering into Warring States Period (475-221BC), with social change accelerated, the traditional ritual was completely destroyed and lead isotope ratios tended to be more scattered over time which indicates the sources of lead ores were diverse [4]. To the Han Dynasty, the manufacture of bronze was not limited to authorities, but more production patterns had emerged such as privately-operated workshops or joint state-private management [1].

From a certain point of view, the political pattern of Western Han Dynasty was stable again just like Zhou Dynasty while great changes had taken place in the model of bronze industry. So what is the situation about the circulation of metal resources at this time? Whether the lead isotope ratios were concentrated as early Spring and Autumn period or decentralized as late Warring States period. What is the nature of the lead ores circulation in the Western Han Dynasty? Is it more like trade pattern or more like distribution pattern? These questions still remains to be studied and discussed.

**Fig 1:**
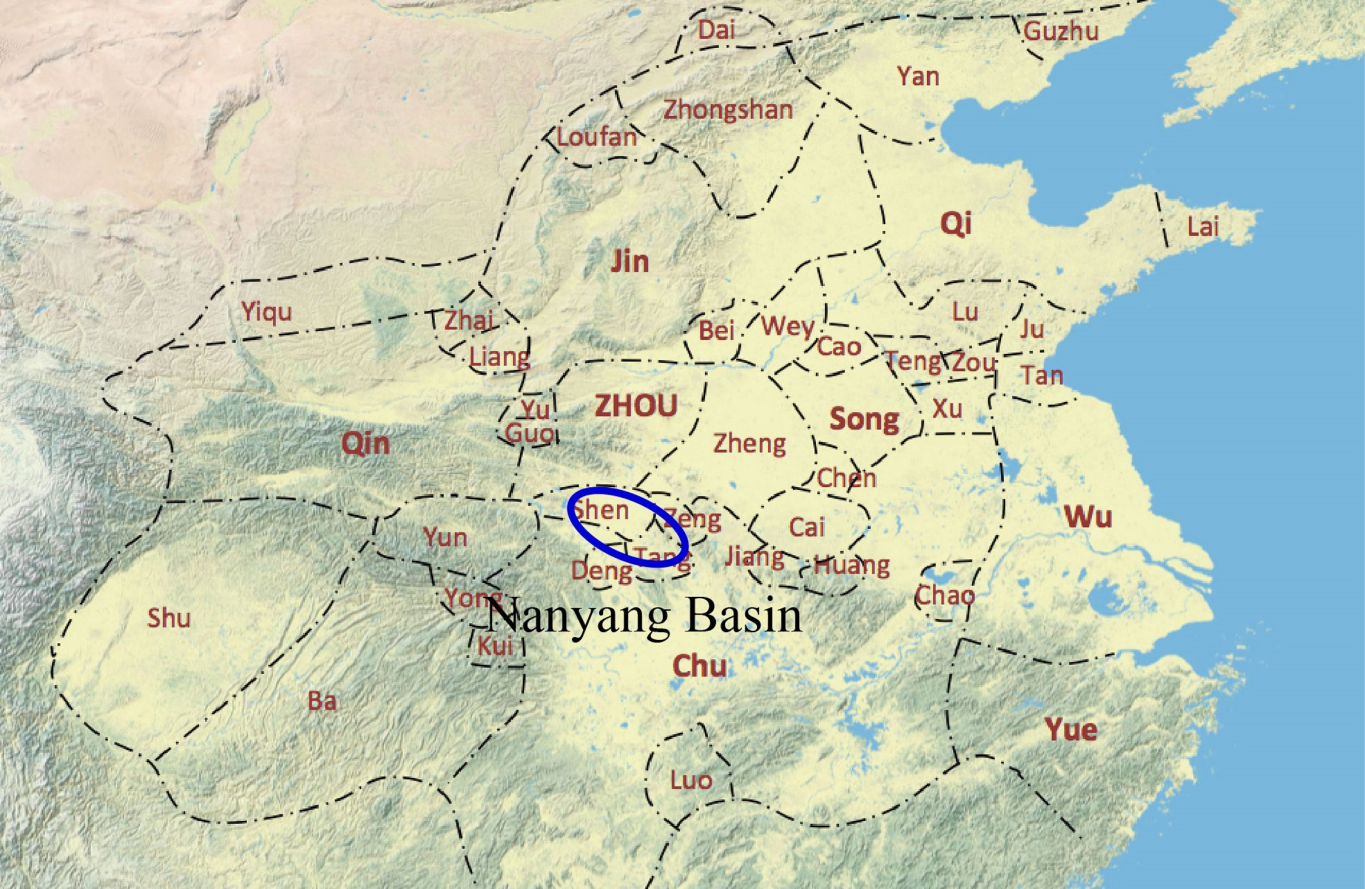
The spatial limits of Nanyang Basin, showed by the blue circle (The background map is the vassal states in the spring and Autumn period).

Nanyang was a famous metropolis in Western Han period and its metallurgy had been continuously developed. More importantly, Nanyang was at the junction of the Central Plains culture and Chu culture, which is an intermontane basin with the characteristics of transitional between northern and southern of China (Fig 1). From the Neolithic Age to the Zhou Dynasty, Nanyang basin had always became the zone of competition between the north and south political power. It was an area easy to communicate and exchange. So it is suitable by taking bronze wares from Nanyang as an example to answer the above questions.

During the Zhou Dynasty, there were many vassal states in the region of present-day Nanyang city, such as E鄂, Shen申, Ying应, Lv吕, Xie谢, Tang唐, Deng邓, Li郦, Liao蓼, Zeng缯, Ruo鄀, etc. [5]. In the late Western Zhou Dynasty, some of the vassal states, for example, Shen and Ying developed its own local bronze culture. The production of bronzes had reached a certain scale in these vassal states and a number of bronzes revealed some peculiar characteristics [6]. In the early ages of the Spring and Autumn Period, the vassal states in Nanyang were successively defeated by the state of Chu, after that Nanyang area was in the control of Chu. The state of Chu had established an administrative region called Wan Yi 宛邑in this place and made it a center of iron smelting [7]. The bronze industry in Nanyang were also inevitably affected by the state of Chu and the style of bronze was changed from the Central Plains culture to the Chu culture [8]. To the Warring States period, the state of Qin occupied Nanyang and set Nanyang County [7]. In the Western Han Dynasty, Nanyang was still famous for its metallurgical industry and gradually developed into a metropolis [9]. The government also set up official agencies in Nanyang to manage bronze wares [1]. Nowadays, increasing number of copper and iron mining sites as well as smelting sites of Han Dynasties were found in Nanyang [10].

### The archaeological samples

Compared with the Warring States period, the burial bronze wares in Han tombs were reduced, not only in quantity but also in types. In the Central Plains, *Ding*, *Fang* and *Shao* were the typical utensils combination always found together in these tombs. The *Ding* vessels are cauldrons, standing upon legs with a lid and two facing handles. The *Fang* vessels are square pots used for wine storage. *Shao* is a spoon made of metal.

**Fig 2:**
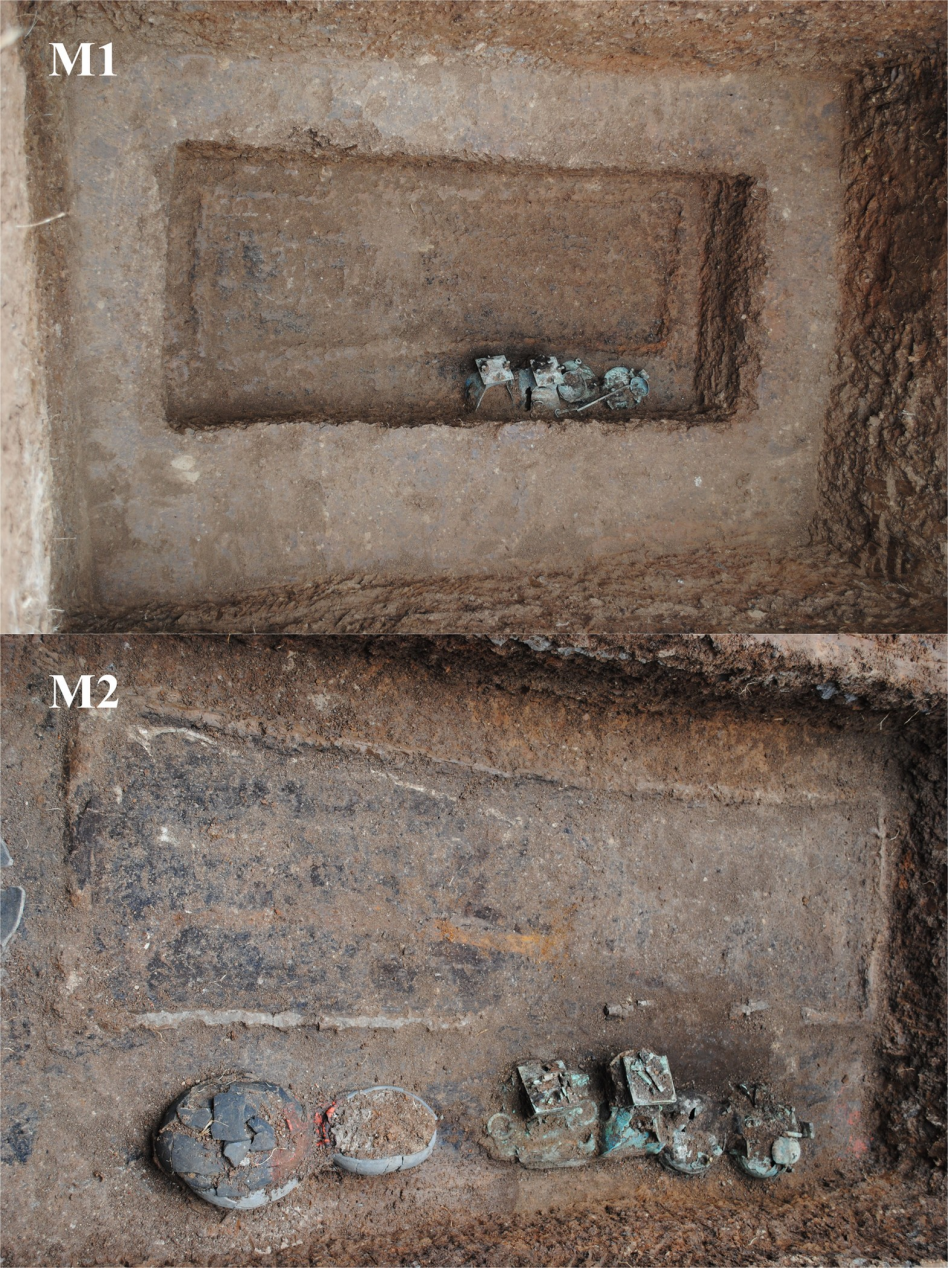
The typical tombs in the Gejiagou site.

Therefore, our study is based on a collection of 14 archaeological bronzes made up of these wares in the Early Western Han Dynasty. The bronze artifacts have been selected from the Gejiagou site. The site is located in the West Bank of the Danjiang Reservoir of the Cangfang Town, Xichuan County in Nanyang City, Henan Province, and was first excavated in the summer of 2016 (Fig 2).

**Fig 3:**
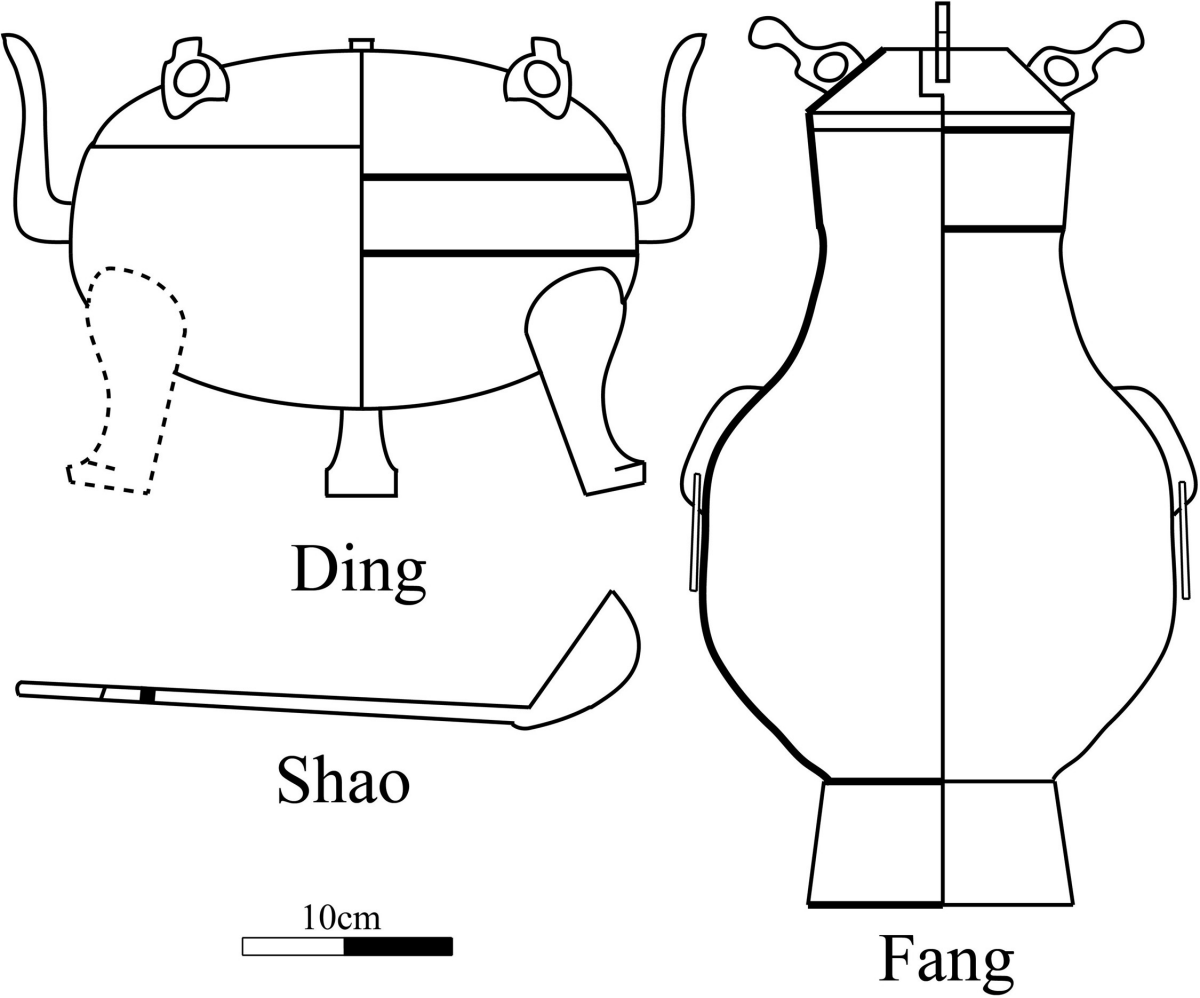
Line drawings of the typical bronze sacrificial wares excavated from the Gejiagou site.

This archaeological excavation was carried out by the Institute of Cultural Relic Research of Nanyang City. Sixty-eight earth-pit tombs and eleven brick tombs were excavated, with more than 300 pieces of pottery, 30 bronze wares, 10 iron wares as well as 4 jade and stone wares. Information about the bronze samples analyzed in this paper was listed in Table 1 and shown in Fig 3.

**Table 1:** Burial units and types of the analyzed bronze artifacts unearthed in Gejiagou.

Lab No.	Burial unit	Type
NY1	M1 1	A piece of a *Fang* vessel
NY2	M1 2	A piece of a *Fang* vessel
NY3	M1 3	A piece of a *Ding* vessel
NY4	M1 4	A piece of a *Ding* vessel
NY5	M2 6	A piece of a *Fang* vessel
NY6	M2 7	A piece of a *Fang* vessel
NY7	M2 8	A piece of a *Ding* vessel
NY8	M2 9	A piece of a *Ding* vessel
NY9	M6 1	A piece of a *Ding* vessel
NY10	M6 2	A piece of a *Ding* vessel
NY11	M26 1	A piece of a *Shao* ladle
NY12	M30 6	A piece of a *Shao* ladle
NY13	M35 12	A piece of a *Shao* ladle
NY14	M38 1	A piece of a *Shao* ladle

Two *Ding* vessels and two *Fang* vessels were respectively unearthed in M1 and M2. They are both earth-pit tombs and regarded as the same couple-burial unit with separate graves by the archaeologist. A pair of *Ding* vessels was from M6 and 4 *Shao* wares were from different tombs respectively. The same utensils were quite common in that time and also found in other places of Nanyang such as Kylin Gang [11]. Owners of these tombs were supposedly the officials and lower classes of aristocracy of the Western Han.

## Analytical methods

### SEM-EDS analysis

Firstly, a small piece of each bronze ware was removed and inserted into a bakelite cylinder by a metallographic inlaying machine. Then, the elemental compositions of the fresh section were determined by a SEM-EDS (HITACHI, TM3030, Japan). In order to reduce the systematic errors, each of these samples was tested three times, and the average value is the final result.

### Lead isotope analysis by MC-ICP-MS

Lead isotope analyses were carried out at School of Archaeology and Museology, Peking University in Beijing, China. Firstly, 2mg of small bronze pieces were chipped off. The pieces were then dissolved in 3 ml of HCl and 1 ml of HNO_3_. Subsequently, the clear solution was leached and diluted with deionized water to 10 ml. The solutions were then measured to detect the lead contents by ICP-AES (PHD, Leeman Labs Inc., California, USA). According to the results representing the lead contents, the solutions were diluted down to 1000ppb. The thallium (T1) standard SRM997 was added in the solutions. Lead isotope analyses were carried out by a MC-ICP-MS (VG AXIOM, Thermo-Elemental Inc., Winsford, England). The spectrometer is a double focusing magnetic sector instrument equipped with an array of 10 variable Faraday collectors. And it has a further fixed Faraday and an electron multiplier detector. Based on repeated analysis of SRM981, the overall analytical 2σ error for all lead isotope ratios was less than 0.238% (Table 2).

**Table 2:** The results of two runs for the SRM981 determination, analytical 2σ error and published values from Cattin et al. [12] are shown.

ICP-MS	SRM981		
Number	^206^Pb/^204^Pb	^207^Pb/^204^Pb	^208^Pb/^204^Pb
1	16.941	15.496	36.718
2	16.945	15.498	36.716
Cattin et al. (2011)	16.942	15.496	36.720
Analytical 2σ error (%)	0.238	0.047	0.072

## Results and discussion

### Alloy compositions and lead isotope ratios

Elemental concentrations of the bronze samples are given in S1 Table. The content of tin in all samples is between the range of 4%~14%. The lead concentrations in almost all investigated samples are between 2% and 4%. The alloy ratio of these bronzes is quite stable. Some scholars think that lead should be introduced by copper ores if the lead content is between 50 ppm and 4% [13]. But in some published literature, a lead amount of more than 2% is considered as an intentional alloy component [14]. Lead may be regarded as an intentional alloying addition even in low amounts, if it would be absent in the original copper ore [15]. Moreover, it is considered that if the lead isotope ratios of a number of low-lead bronze wares at the same archaeological site are very close, the lead should be introduced in copper ores [16]. Contrary to this view, the lead isotope ratios of Gejiagou bronzes are not very concentrated. On the base of the above, all the samples were Cu-Sn-Pb ternary alloy. It suggests the lead should be added on purpose.

Therefore, it was confirmed that the lead isotopes point to the source of lead ores. S2 Table shows that the lead isotope ratios conform to the characteristics of the common lead [17]. According to the mapping of geochemical provinces in China based on the lead isotopic compositions, there are six geochemical provinces as follows: Northern China, Yangtze, southern China, Northern Xinjiang, Jiamusi and Tibet [18]. We find that NY9 and NY13 drop into the region of southern China and other samples are from Northern China. It seems that these samples may have at least two different lead ore sources and they have some distance in geographical locations.The published isotope data of bronze wares in the Eastern Zhou Dynasty (c.770–256 BC) can be roughly divided into three classes [4]. Crudely put, Class A appeared in the early ages of the spring and Autumn Period (7th Century BC) with ^208^Pb/^206^Pb ratios between 2.11 to 2.13 and ^207^Pb/^206^Pb ratios from 0.85 to 0.8; Class B was popular in the middle of the spring and Autumn Period (6th Century BC) with ^208^Pb/^206^Pb ratios between 2.09 to 2.11; Class C predominated in Central China since the 5th Century BC characterized by ^208^Pb/^206^Pb ratios between 2.16 to 2.18. Moreover, the lead isotope ratios became much more scattered during the Warring States period.

This conclusion was also verified by the lead isotopic study of bronze in Nanyang. Some bronze artifacts of the early E state in the Zhou Dynasty (7th Century BC) showed that all the lead mineral used in this region belonged to Class A [19]. But another group of bronzes of Shen County, which could be dated back to the late Spring Autumn period and the early Warring States period (c.516–256 BC), displayed a large range of lead isotope data across all three classes [20].

**Fig 4:**
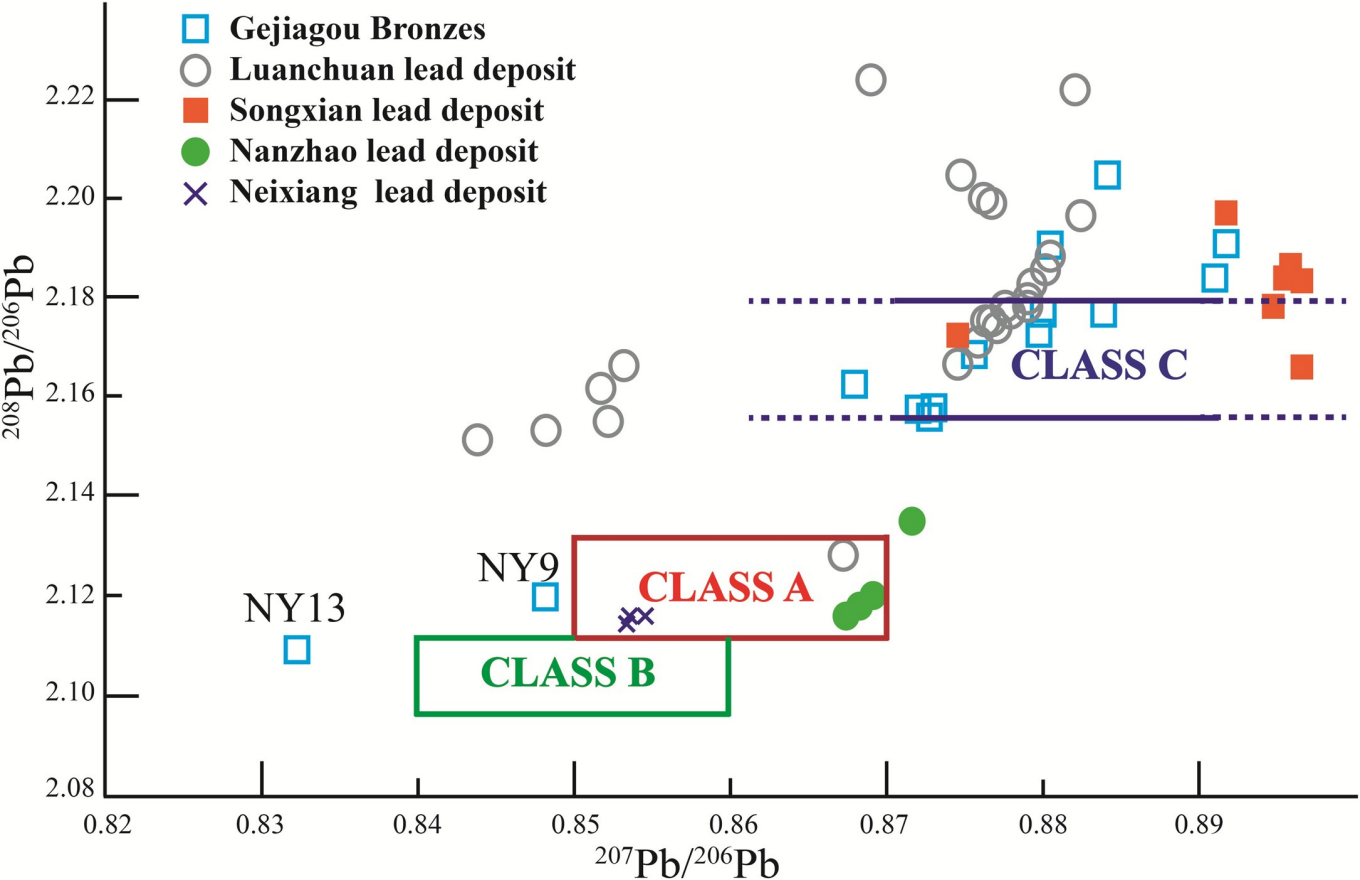
Lead isotope ratios of Gejiagou bronzes and lead deposits in the surrounding area of the Nanyang Basin.

However, after Warring States period, the situation in Nanyang became more complex. The lead isotope ratios of Gejiagou bronzes can not be completely covered by three classes (Fig 4 and S3 Table). Although most of bronze wares in the Gejiagou Site used the lead minerals of Class C, NY9 and NY13 did not. NY9 is closer to Class A while NY13 to Class B. Noticing that all of the known lead isotope ratios ^207^Pb/^206^Pb in Central China are higher than 0.84, NY13 is obviously different from these three classes. The increasing number of lead ore resources is an epitome of the prosperity of the metallurgical industry in Nanyang.

### Lead ore sources

The metallurgy in Nanyang was relatively advanced from ancient times. Some copper casting sites of Han Dynasty were still found in places like Nanzhao County, Tongbai County and Wancheng [10]. There are also many galena resources in Nanyang and its surrounding areas, such as Luchuan County [21], Songxian County [22], Nanzhao County [23], Neixiang County [24] and so on. Although these places are geographically close, the lead isotope ratios vary in different locations because they are on the belt of southern Qinling Mountains (S3 Table). Lead minerals from Nanzhao and Neixiang can cover Class A while lead minerals from Luanchuan and Songxian can encompass Class C. In other words, both two classes of lead ores can be found in Nanyang area. Therefore, all bronzes from the Gejiagou Site except NY9 and NY13 very likely used local lead minerals in Nanyang.

**Fig 5:**
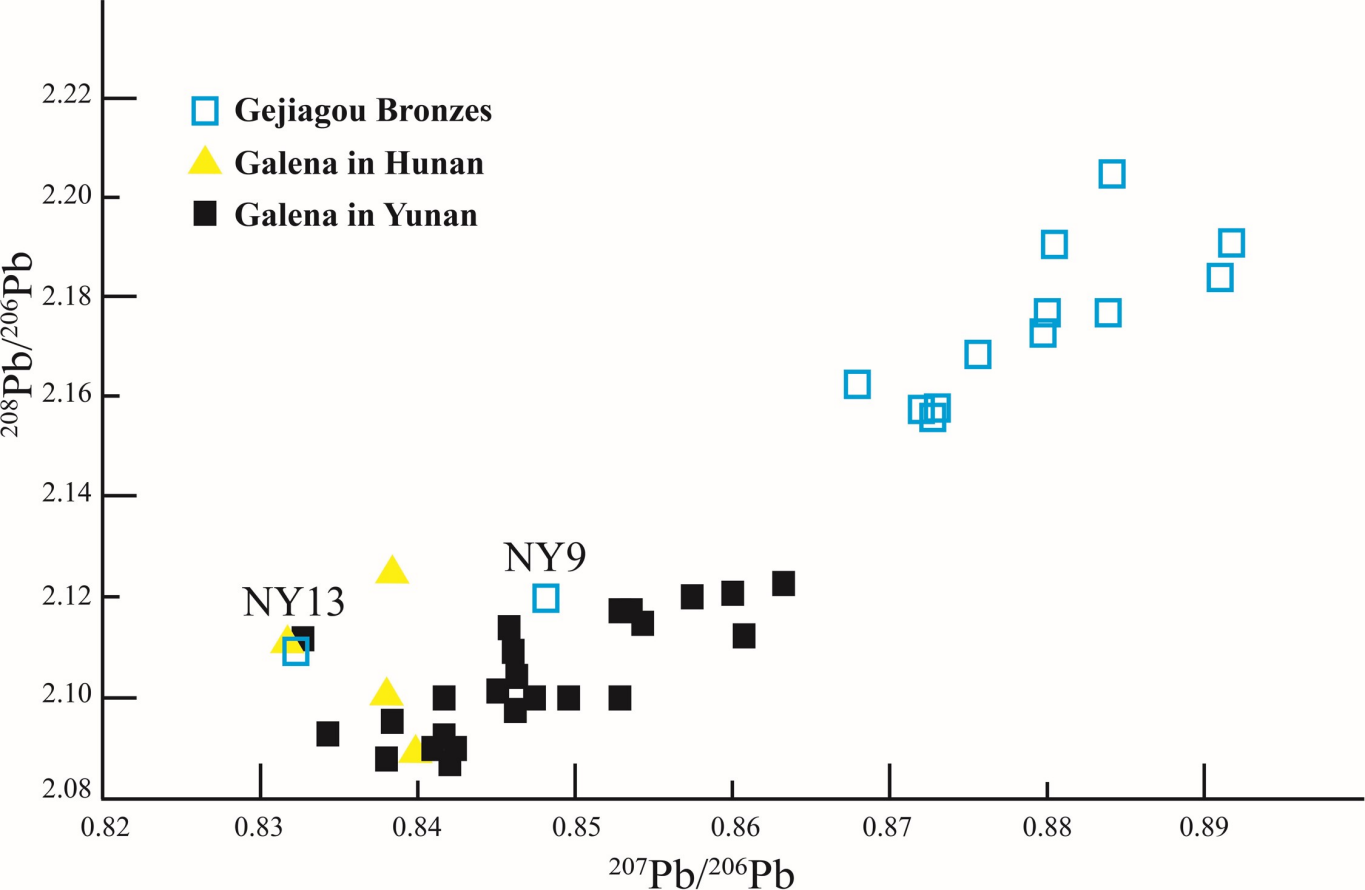
Lead isotope ratios of Gejiagou bronzes and galena in Hunan and Yunnan provinces.

The lead source of these two bronzes needs to be discussed. As mentioned earlier, according to the geochemical provinces, NY13 belongs to the region of southern China and NY9 close to this region. We found in Yunnan, Hunan, Guangxi and Sichuan provinces existed galena similar to NY9 and NY13 [25, 26, 27, 28]. Here, we have collected the isotope ratios of lead ore in central Yunnan and central Hunan due to stronger similarity (S3 Table). In Fig 5, there are two data from different places almost coinciding with NY13 respectively and NY9 was surrounded by the data of Yunnan. Moreover, the lead ratio of NY9 can be obtained by linear combination of Yunnan galena. Anyhow, lead minerals of NY13 and NY9 were not from Central China but some places further south.

Before this period, the lead isotopes of bronzes in Nanyang could be generally reached with the local galena. However, sufficient lead materials did not limit people's access to the same resource from other places. The range of lead ores circulation in Central China had become wider and longer during the early Western Han Dynasty. Certainly, the raw ores would be processed, refined and then transported.

More importantly, refining galena into lead ingots was quite common in central Hunan during the Spring and Autumn period and the Warring States Period. For example, in Changsha and Xiangtan, some lead ingots with high purity were unearthed [29, 30]. Combined with so many lead mineral resources in Hunan, local lead smelting industry should be more developed in the Western Han Dynasty. Therefore, we speculate that there was a road of metal resources from southern China to Central China.

### Lead ore circulation

In the Western Han Dynasty, the transportation in Central China were much developed. Great progress had been made in many aspects such as road construction, water transportation, vehicle technology, transport power, traffic settings, etc. [31]. However, based on ‘Historical Records Huo Zhi Biography’, southern China had a sparse population and the economy was also less developed than Central Plains at that time. Moreover, there are almost no direct literatures about the transportation in these regions.

According to Historical Records, Liu Fa (?-129 BC), the King of Changsha in the Western Han Dynasty, would sent fine rice to his mother in Chang’an from Changsha every year. At the same time, the soil in Chang’an would be transported back to Changsha and be used to build a platform. Therefore, the traffic network was able to reach southern China and the interaction between the two regions was more frequent.

Moreover, also according to Historical Records, Emperor Wu conquered the Yunnan area and granted the local king a golden seal, which was unearthed at the Shizhaishan site in 1956 [32]. In the Western Han Dynasty, the central government set up prefectures and counties in Yunnan. A large number of bronze and iron objects with Central Plains culture were found in tombs of this area, such as bronze mirrors, bronze trappings, agricultural production tools and so on. For example, there is a bronze crossbow with inscriptions saying that it was made in the Central Plains. These archaeological evidence indicates that there was a stable road from southern China to central China at that time [33].

Specifically, here are some reasons to explain the communication between Nanyang and southern China. First of all, located in Central China, Nanyang linked a lot of other areas in Western Han Dynasty as a transportation hub. There were at least five traffic arteries on land and numerous waterways. Secondly, after Qin occupied Nanyang in the late Warring States Period, a large number of immigrants were forced to move into Nanyang, among them many were merchants and handicraft industry practitioners. Because of the long and large scale of the migrating activities, a lot of materials and technologies had been brought to Nanyang [9]. Thirdly, the Western Han Dynasty ushered in the era of peace after many years of war and recuperating is the main policy in this stage. The state had liberalized the power of resources utilization to the public. Specifically, the enlightened administration of the Han emperors Wen and Jing pursued a liberal policy and played a series of positive roles on the country's economic recovery, capital construction, public education and so on [9]. As a result, many individual workshops also including metallurgical workshops were everywhere.

For this reason, the more scattered lead isotope ratios of Gejiagou bronzes indicated they might come from different workshops. The circulation of lead ore is entirely the result of free trade. However, even if the local resources were rich, there was still a exchange of external resources. Hereafter the range of lead ore circulation become much wider.

## Conclusions

In the paper, fourteen bronze samples from the Gejiagou site, Nanyang city, Henan Province, which dated back to the early Western Han Dynasty, the tail end of the bronze period, were analyzed for elemental concentrations and lead isotope ratios.

The elemental concentrations show that the alloy ratios are very stable. It also shows that the lead should be introduced on purpose, and the lead isotopes indicate the provenance information of lead ores. The lead isotope ratios conform to the characteristics of common lead, which are the same as the bronze wares of the Eastern Zhou Dynasty in Central China.

All these samples may have more than single lead ore sources different from previous situation. Most of bronze wares in the Gejiagou Site used the local lead mineral. But another two of them, NY9 and NY13 used lead ores from southern China. The range of lead ore circulation become much wider due to the loose policy, economic prosperity, transportation improvement and so on. It was not just local and regional circulation like before but already changed into a nationwide trade.

## Supporting information

S1 Table Elemental compositions for the artifacts studied. (PDF)

S2 Table Lead isotope ratios for the artifacts studied. (PDF)

S3 Table Lead isotope ratios of the lead ores mentioned in this article. (PDF)
