# Supplementary material for: The lead ores circulation in Central China during the early Western Han Dynasty: A case study with bronze vessels from the Gejiagou site
Source: PLoS One. 2018 Nov 7;13(11):e0205866. doi: 10.1371/journal.pone.0205866 (PMC6221295; doi:10.1371/journal.pone.0205866)
Supplement: S1 Table — (PDF) [file pone.0205866.s001.pdf]

S1 Table. Elemental compositions for the artifacts studied.

Lab No.	Elemental concentration (wt%)			
	Cu	Sn	Pb	Total
NY1	79.04	13.46	3.74	96.24
NY2	80.28	12.63	3.26	96.17
NY3	83.19	10.43	2.43	96.05
NY4	79.67	12.31	2.12	94.1
NY5	80.32	11.41	2.75	94.48
NY6	79.81	10.98	3.96	94.75
NY7	78.09	12.88	3.27	94.24
NY8	79.38	11.94	4	95.32
NY9	78.13	11.96	4.14	94.23
NY10	79.82	10.39	3.63	93.84
NY11	90.6	4.38	3.18	98.16
NY12	87.3	6.85	3.75	97.9
NY13	88.19	6.92	2.41	97.52
NY14	89.61	4.8	3.2	97.61
