# Supplementary material for: The lead ores circulation in Central China during the early Western Han Dynasty: A case study with bronze vessels from the Gejiagou site
Source: PLoS One. 2018 Nov 7;13(11):e0205866. doi: 10.1371/journal.pone.0205866 (PMC6221295; doi:10.1371/journal.pone.0205866)
Supplement: S2 Table — (PDF) [file pone.0205866.s002.pdf]

S2 Table. Lead isotope ratios for the artifacts studied.

Lab No.	Lead isotope ratios				
	$^{206}Pb/^{204}Pb$	$^{207}Pb/^{206}Pb$	$^{208}Pb/^{206}Pb$	$^{207}Pb/^{204}Pb$	$^{208}Pb/^{204}Pb$
NY1	17.7270	0.8757	2.1692	15.5239	38.4539
NY2	17.5031	0.8839	2.1765	15.4718	38.0954
NY3	17.7934	0.8726	2.1570	15.5268	38.3807
NY4	17.8009	0.8724	2.1575	15.5287	38.4048
NY5	17.8033	0.8723	2.1564	15.5291	38.3915
NY6	17.3166	0.8911	2.1840	15.4315	37.8200
NY7	17.6171	0.8797	2.1727	15.4985	38.2812
NY8	17.3079	0.8918	2.1904	15.4350	37.9105
NY9	18.4563	0.8482	2.1194	15.6550	39.1165
NY10	17.6240	0.8795	2.1767	15.5003	38.3613
NY11	17.9263	0.8680	2.1624	15.5609	38.7650
NY12	17.5414	0.8842	2.2046	15.5098	38.6720
NY13	18.8345	0.8323	2.1087	15.6757	39.7141
NY14	17.6022	0.8806	2.1901	15.4998	38.5545
