# Supplementary material for: The lead ores circulation in Central China during the early Western Han Dynasty: A case study with bronze vessels from the Gejiagou site
Source: PLoS One. 2018 Nov 7;13(11):e0205866. doi: 10.1371/journal.pone.0205866 (PMC6221295; doi:10.1371/journal.pone.0205866)
Supplement: S3 Table — (PDF) [file pone.0205866.s003.pdf]

S3 Table. Lead isotope ratios of the lead ores mentioned in this article.

No.	$^{207}Pb/^{206}Pb$	$^{208}Pb/^{206}Pb$	Location
LC01	0.8532	2.1665	Luanchuan
LC02	0.8766	2.1993	Luanchuan
LC03	0.8762	2.1997	Luanchuan
LC04	0.8482	2.1531	Luanchuan
LC05	0.8517	2.1617	Luanchuan
LC06	0.8769	2.1755	Luanchuan
LC07	0.8439	2.1511	Luanchuan
LC08	0.8522	2.1552	Luanchuan
LC09	0.8783	2.1764	Luanchuan
LC10	0.8783	2.1765	Luanchuan
LC11	0.8807	2.1880	Luanchuan
LC12	0.8794	2.1771	Luanchuan
LC13	0.8795	2.1836	Luanchuan
LC14	0.8770	2.1741	Luanchuan
LC15	0.8775	2.1755	Luanchuan
LC16	0.8782	2.1770	Luanchuan
LC17	0.8802	2.1857	Luanchuan
LC18	0.8782	2.1762	Luanchuan
LC19	0.8776	2.1736	Luanchuan
LC20	0.8761	2.1719	Luanchuan
LC21	0.8792	2.1813	Luanchuan

LC22	0.8746	2.1666	Luanchuan
LC23	0.8783	2.1759	Luanchuan
LC24	0.8779	2.1776	Luanchuan
LC25	0.8673	2.1280	Luanchuan
LC26	0.8789	2.1796	Luanchuan
LC27	0.8825	2.1966	Luanchuan
LC28	0.8748	2.2045	Luanchuan
LC29	0.8691	2.2239	Luanchuan
LC30	0.8821	2.2218	Luanchuan
SX01	0.8947	2.1785	Songxian
SX02	0.8966	2.1657	Songxian
SX03	0.8966	2.1841	Songxian
SX04	0.8918	2.1973	Songxian
SX05	0.8957	2.1833	Songxian
SX06	0.8960	2.1851	Songxian
SX07	0.8744	2.1725	Songxian
NZ01	0.8682	2.1168	Nanzhao
NZ02	0.8676	2.1151	Nanzhao
NZ03	0.8717	2.1344	Nanzhao
NZ04	0.8677	2.1149	Nanzhao
NX01	0.8545	2.1152	Neixiang
NX02	0.8536	2.1159	Neixiang
NX03	0.8535	2.1137	Neixiang
YN01	0.8674	2.1170	Yunan

YN02	0.8402	2.0887	Yunan
YN03	0.8378	2.0876	Yunan
YN04	0.8458	2.1132	Yunan
YN05	0.8691	2.1136	Yunan
YN06	0.8528	2.1167	Yunan
YN07	0.8421	2.0907	Yunan
YN08	0.8475	2.0996	Yunan
YN09	0.8526	2.1007	Yunan
YN10	0.8463	2.0965	Yunan
YN11	0.8450	2.1009	Yunan
YN12	0.8628	2.1225	Yunan
YN13	0.8417	2.0925	Yunan
YN14	0.8326	2.1090	Yunan
YN15	0.8416	2.0900	Yunan
YN16	0.8422	2.0873	Yunan
YN17	0.8393	2.0885	Yunan
YN18	0.8277	2.0649	Yunan
YN19	0.8608	2.1112	Yunan
YN20	0.8417	2.0990	Yunan
YN21	0.8446	2.0789	Yunan
YN22	0.8493	2.0991	Yunan
YN23	0.8740	2.1232	Yunan
YN24	0.8687	2.1191	Yunan
HN01	0.8380	2.1254	Hunan

HN02	0.8321	2.1107	Hunan
HN03	0.8400	2.0851	Hunan
HN04	0.8386	2.1085	Hunan
